# The Interplay of Protein Hydrolysis and Ammonia in the Stability of *Hevea* Rubber Latex during Storage

**DOI:** 10.3390/polym15244636

**Published:** 2023-12-07

**Authors:** Narueporn Payungwong, Jitladda Sakdapipanich, Jinrong Wu, Chee-Cheong Ho

**Affiliations:** 1 Department of Chemistry and Center of Excellence for Innovation in Chemistry, Faculty of Science, Mahidol University, Nakhon Pathom 73170, Thailand; narueporn.pau@student.mahidol.ac.th; 2 State Key Laboratory of Polymer Materials Engineering, College of Polymer Science and Engineering, Sichuan University, Chengdu 610065, China; wujinrong@scu.edu.cn; 3 Faculty of Science, University Tunku Abdul Rahman, Sungai Long Campus, Kajang 43000, Malaysia; cchoho2001@yahoo.com

**Keywords:** *Hevea brasiliensis*, natural rubber latex, ammonia preservation, protein hydrolysis, spatial distribution of allergenic proteins in NR latex, deproteinized latex, latex film formation, film morphology changes during storage

## Abstract

Natural rubber (NR) latex derived from *Hevea brasiliensis* is a complex colloid comprising mainly rubber hydrocarbons (latex particles) and a multitude of minor non-rubber constituents such as non-rubber particles, proteins, lipids, carbohydrates, and soluble organic and inorganic substances. NR latex is susceptible to enzymatic attack after it leaves the trees. It is usually preserved with ammonia and, to a lesser extent, with other preservatives to enhance its colloidal stability during storage. Despite numerous studies in the literature on the influence of rubber proteins on NR latex stability, issues regarding the effect of protein hydrolysis in the presence of ammonia on latex stability during storage are still far from resolved. The present work aims to elucidate the interplay between protein hydrolysis and ammoniation in NR latex stability. Both high- and low-ammonia (with a secondary preservative) NR latexes were used to monitor the changes in their protein compositions during storage. High-ammonia (FNR-A) latex preserved with 0.6% (*v*/*v*) ammonia, a low 0.1% ammonia/TMTD/ZnO (FNR-TZ) latex, and a deproteinized NR (PDNR) latex were labeled with fluorescence agents and observed using confocal laser scanning microscopy to determine their protein composition. Protein hydrolysis was confirmed via sodium dodecyl sulfate–polyacrylamide gel electrophoresis (SDS-PAGE). The results revealed that protein hydrolysis increased with the storage duration. The change in protein composition accompanying hydrolysis also allows the spatial distribution of allergenic proteins to be estimated in the latex. Concurrently, the latex stability increased with the storage duration, as measured by the latex’s mechanical stability time (MST) and the zeta potential of the latex particles. As monitored by AFM, the surface roughness of the NR latex film increased markedly during extended storage compared with that of the DPNR latex, which remained smooth. These results underscore the pivotal role of ammonia in bolstering NR latex stability brought on by protein hydrolysis, which greatly impacts latex film’s formation behavior. NR latex stability underpins the quality of latex-dipped goods during manufacturing, particularly those for medical gloves.

## 1. Introduction

Natural rubber (NR) latex, tapped from *Hevea brasiliensis*, is a colloidal system composed of mostly spherical latex particles [1]. Freshly tapped NR (FNR) latex contains approximately 30–40% rubber hydrocarbons, namely, *cis*-1,4-polyisoprene, and 5% non-rubber components, which are 1.4% proteins, 1.0% lipids, 0.6% phospholipids, and 0.5% ash [2,3]. The surface of the rubber particles is covered with proteins, phospholipids, and lipids in a mixed monolayer that stabilizes the latex [4,5,6]. Rubber molecules are known to have two terminal chain ends, of which the α-end is associated with phospholipids and the ω-end is associated with proteins [3,7,8,9,10,11,12,13,14]. After leaving the trees, if the latex is not preserved, its pH will drop due to the formation of volatile fatty acids (VFAs) following biochemical reactions, leading to putrefaction and coagulation [15]. Therefore, the NR latex is usually stabilized with ammonia solution, and possibly with other secondary preservatives, before being transported from the plantation to the latex concentrate factory to produce commercial ammoniated NR latex concentrate containing 60% *w*/*w* rubber. The ammonia content is typically raised to 0.6–0.7% for the long-term preservation of concentrated latex, referred to as “high-ammonia preserved concentrated NR latex (HA)”. Another type is called “low-ammonia preserved concentrated NR (LA-TZ) latex”, which contains only 0.1% ammonia together with tetramethylthiuram disulfide (TMTD) and zinc oxide (ZnO) as bactericides. Ammonia is a primary preservative, while also functioning as a bactericide for microorganisms to enhance the colloid stability of the latex [16,17]. During storage under high-ammonia conditions, negatively charged long-chain fatty acid soaps resulting from the hydrolysis of unbound polar lipids at the rubber particle surface become adsorbed and maintain the stability of the latex [18,19,20]. On the other hand, rubber proteins are essential components of NR latex that are responsible for the biosynthesis of rubber molecules and strongly affect their physical and colloidal properties [21]. Several proteins in NR have been found to cause type I allergic responses that can lead to life-threatening anaphylactic reactions in sensitized individuals [22,23,24,25]. The total protein content of FNR latex is approximately 1–1.5%, of which about 27% is associated with the rubber particles. A similar amount is associated with the bottom fraction, with the remainder dissolved in the serum phase [26]. The proteins associated with the rubber molecules are at the ω-terminus, and its ammonia-induced negative charge contributes to the colloidal stability of the latex [3,27]. However, there is a general lack of information on the protein composition of ammoniated latex with respect to storage duration and their spatial locations in the latex.

Commercial HA and LA-TZ latexes are important feedstocks for manufacturing latex-dipped goods, such as medical gloves, catheters, tubing, condoms, dental dams, foams, and mattresses. Stringent leaching protocols are usually included in the manufacturing process to remove residual chemicals used for compounding the latex, and to remove the non-rubbers from the latex. This is particularly important in the case of medical device manufacturing. The leaching process removes most of the soluble protein residues and chemicals; some proteins associated with the rubber may remain. Hence, an in-depth understanding of the hydrolysis process of proteins in ammoniated latex during storage would provide essential information on protein compositions and their location in the latex that could facilitate improved protein leaching of dipped products. This will lead to low-allergenic medical products.

The present study delves into the intricate interplay between ammonia preservation and protein hydrolysis in *Hevea* rubber latex. Our investigation focused on protein composition changes in latex preserved with ammonia under different conditions. Three different types of NR latexes were investigated. The first type was latex preserved with a high concentration of ammonia (0.6% *v*/*v*), the second type was latex preserved with a lower concentration of ammonia (0.1% *v*/*v*) in conjunction with TMTD/ZnO, and the third type was purified NR latex obtained via enzymatic deproteinization with a much-reduced protein content. The changes in protein composition for each latex were determined by fluorescence labeling and visualized by confocal laser scanning microscopy (CLSM). The changes in the protein composition of the latex during storage were further confirmed using sodium dodecyl sulfate–polyacrylamide gel electrophoresis (SDS-PAGE). The allergenic ones were identified, including the smaller hydrolyzed fragments and their locations in the latex. The impact of protein composition changes with storage duration on the films formed by the various latexes was evaluated by atomic force microscopy (AFM). Data on the mechanical stability time (MST) and zeta potential of the latex were used to assess its stability.

## 2. Materials and Methods

### 2.1. Materials

The reagents were supplied or purified according to standard procedures where necessary. AR-grade solvents were used without further purification. Freshly tapped NR latex (FNR), obtained from *Hevea brasiliensis* (clone RRIM 600), was provided by the Thai Rubber Latex Corporation (Samutprakan, Thailand) Public Co., Ltd. The FNR was concentrated by centrifugation at a rotation speed of 8000 rpm for 30 min and preserved with ammonia immediately: (i) 0.6% *v*/*v* ammonia, and (ii) 0.1% *v*/*v* ammonia with 0.025% *w*/*v* tetramethylthiuram disulfide (TMTD) and 0.025% *w*/*v* zinc oxide (ZnO), to produce FNR-A and FNR-TZ latexes, respectively. Deproteinized NR latex was prepared from the FNR latex by incubating it with 0.04% *w*/*v* proteolytic enzyme (KP 3939, Kao Co., Tokyo, Japan) and 0.2% *w*/*v* Triton^®^X-100 at 37 °C for 24 h, followed by centrifugation at 20,292× *g* for 1 h. The cream fraction was collected and redispersed in Triton^®^X-100 at 0.2% *w*/*v* and then centrifuged again. The cream fraction was adjusted to 60% dry rubber content (DRC) with 0.2% *w*/*v* Triton^®^X-100.

### 2.2. Determination of Zeta Potential

A latex sample (100 µL) was added to deionized water (type 1, 20 mL). The resulting mixture was adjusted to pH 10 using a 0.1% *w*/*v* potassium hydroxide (KOH) solution. This sample was injected into the sample cell of the Malvern Autoanalyzer 4700 (Malvern Panalytical Ltd., Malvern, UK) to be cleaned by flushing. The zeta potential was then measured at 25 °C, with the dielectric constant set at 79, cell field at 28.9 V/cm, and current at 1.5 mA.

### 2.3. Determination of Mechanical Stability Time (MST)

The latex stability was determined using an MST machine operated by Unitronics Vision 120TM (Unitronics Inc., Quincy, MA, USA). A latex sample adjusted to 30% total solid content (approx. 80 g) was sheared under high-speed agitation (14,000 rpm) at 35 °C until the first sign of flocculation in seconds (s).

### 2.4. Fluorescence Labeling and Analysis of NR Particles Using Confocal Laser Scanning Microscopy (CLSM)

This non-invasive technique allows us to examine individual latex particles at a constant magnification and wavelength ratio [18,28]. In this study, we used fluorescent dye specifically for labeling proteins to assess its 3D distribution on the surface of the latex particles. This approach can provide high lateral (x–y) and axial (z) resolution. This enables the quantification of proteins associated with each particle. The method proves valuable for comparing the levels of surface proteins on various latex types as they undergo aging during storage. However, no data on internal composition are available, because the resolution of CLSM used is not high enough for such determination. The experimental details of the labeling procedure and fluorescence measurement are given below.

FNR latex (1 mL) was centrifuged at 20,292× *g* to remove the serum. The obtained cream fraction was redispersed in deionized water (type 1, 1 mL) and incubated with a Qubit™ Protein Assay Kit (Thermo Fisher Scientific Inc., Waltham, MA, USA). The Qubit working solution was prepared by diluting the Qubit protein reagent A at a ratio of 1:200 in Qubit protein buffer in the assay tubes, and then vortexed. All of the fluorescence-labeled latex samples were stored in a dark container at 16 °C overnight. The excess dye was removed using a dialysis membrane with a molecular weight cutoff of 50 kDa for 6 h. The resulting RP sample was deposited on a glass slide and covered with a coverslip.

Fluorescent images of the RPs were captured using a confocal laser scanning microscope Olympus FV-1000 (Olympus Singapore PTE Ltd., Singapore) equipped with four laser systems (Multi AR, HeNe-G, HeNe-R, and LD405/440 laser diode) and a transmitted light detector with an oil-immersion objective lens (60×), and then the images were processed using an integrated image analysis program (Olympus Fluoview). The optimal sampling z-thickness was fixed at 550 nm/slice. Different filter set combinations, TRITC, and Alexa Fluor 633 (Thermo Fisher Scientific Inc., Waltham, MA, USA) were used to measure the fluorescence emission intensities of Qubit-dye-bound proteins on the NR particles (determined from the images and subtracted from the background). Each value of the obtained fluorescence intensity was divided by the cross-sectional area of each particle: ∆FI_particle_ = (FI_NR_ − FI_background_)/area of a particle (µm^2^). The value was then averaged with the other particles in the sample.

The fluorescence spectra were recorded using a JASCO FP-6200 spectrofluorometer (JASCO International Co., Ltd., Tokyo, Japan) using a Xe lamp as a light source, a silicon photodiode for the excitation monochromator, and a photomultiplier for the emission monochromator. Qubit protein was approximated to the fluorescence excitation/emission maximum wavelength at 470/570 nm. The corresponding fluorescence was collected in the range of 480–700 nm.

### 2.5. Determination of Proteins

The protein composition of the latex during storage was determined using sodium dodecyl sulfate–polyacrylamide gel electrophoresis (SDS-PAGE). First, small pieces of dried FNR were immersed in a 2% (*w*/*v*) sodium dodecyl sulfate solution to extract the proteins by stirring them at room temperature for 12 h [29]. The mixture was filtered through filter paper to obtain a serum fraction containing water-soluble proteins. The serum proteins were precipitated using a 10% (*w*/*v*) solution of trichloroacetic acid in acetone and a 0.07% (*v*/*v*) aqueous 2-mercapto-ethanol. The mixture was allowed to stand at −20 °C for 45 min. The precipitated proteins were collected by centrifugation at 20,000 rpm at 4 °C for 1 h and then analyzed by sodium dodecyl sulfate–polyacrylamide gel electrophoresis (SDS-PAGE). Prior to this analysis, the protein contents were determined by Bradford micro-assay. The protein analysis was calibrated using standard bovine serum albumin by plotting the average absorbance at 595 nm on a Spectra Max spectrophotometer. For the SDS-PAGE analysis, a 12.5% acrylamide separating gel and a 5% acrylamide stacking gel were used. To prepare the separating gel, all of the ingredients were mixed (i.e., ultrapure (type 1) water, 3.2 mL; 1.5 M Tris-HCl (pH 8.8), 2.5 mL; acrylamide stock solution (30% acrylamide, 0.8% bis-acrylamide), 4.2 mL; 10% sodium dodecyl sulfate, 100 μL; 10% ammonium persulfate, 50 μL; and tetramethylethylenediamine, 3.4 μL). Then, the mixture was injected into a gel electrophoresis cell and kept at room temperature for 30 min to polymerize. All of the ingredients for preparing the stacking gel were mixed using the same method (i.e., ultrapure (type 1) water, 1.7 mL; 1.5 M Tris-HCl (pH 8.8), 0.75 mL; acrylamide stock solution (30% acrylamide, 0.8% bis-acrylamide), 0.5 mL; 10% sodium dodecyl sulfate, 30 μL; 10% ammonium persulfate, 30 μL; and tetramethylethylenediamine, 2 μL). The stacking gel was overlaid on the separating gel, and a well-forming comb was inserted. It was kept at room temperature for 30 min. After the stacking gel had been formed, pre-stained protein standards (Bio-Rad^®^, Hercules, CA, USA, 5 µg) and the samples (5 µg) were separately loaded in the wells. A running buffer (2% sodium dodecyl sulfate solution, 50 mM Tris-HCl buffer solution, 6 M urea solution, 30% glycerol solution, and 0.002% bromophenol blue solution) was added to the electrophoresis system. The proteins were separated by applying a voltage of 120 V. After electrophoresis, the gel was stained with Coomassie brilliant blue R-250 for 24 h and washed several times with ultrapure water. The gel thus obtained was imaged using an Image Scanner II and analyzed using ImageMaster 2D Platinum software v7.0.

### 2.6. Characterization of Surface Morphology

Latex-dipped films were prepared by dipping clean glass plates into 50 mL of latex samples with a 10 wt.% dry rubber. Then, the latex films were purged with nitrogen gas for 5 min and dried at room temperature (25 ± 3 °C) for 15 min. The prepared films were then kept in desiccators for various durations (1, 7, 14, and 21 days) before viewing them with the AFM.

The surface topography of the dipped films was examined using a NanoScope III (Digital Instruments, Santa Barbara, CA, USA) equipped with a J scanner for a maximum scan area of 150 μm^2^. The morphological images were recorded on a 5 × 5 µm^2^ scan area using the tapping mode. The surface roughness of each film sample was represented by the average surface roughness (Ra), which was calculated from three different areas of interest on the film surface. The Ra value of each scanning area was calculated using $R_{a}=(\frac{1}{L})\int_{0}^{L}\left|Z(x)\right|\mathrm{dx}$, where L is the evaluation length and Z(x) is the profile height function.

## 3. Results

### 3.1. Characterization of Latexes

Some results of the mean diameters, protein contents, and associated fatty acid ester contents of the FNR-A, FNR-TZ, and DPNR latexes are presented in Table 1. A comparison revealed hardly any significant changes in these basic properties of the latexes, except that the protein contents of the DPNR latex were significantly reduced due to deproteinization. However, the ester content and the mean particle size of DPNR remained comparable to those of both other FNR samples.

**Figure 1:**
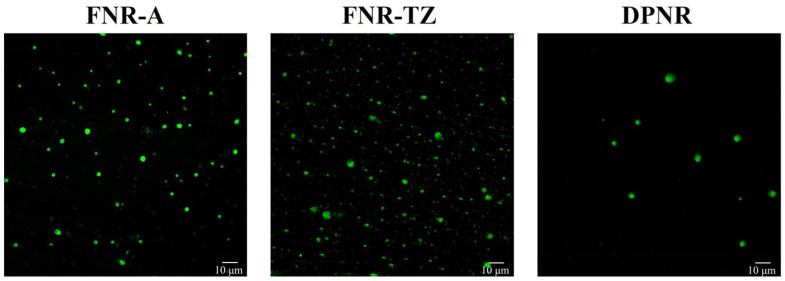
The fluorescence images of FNR-A, FNR-TZ, and DPNR obtained via confocal laser scanning microscopy.

**Figure 2:**
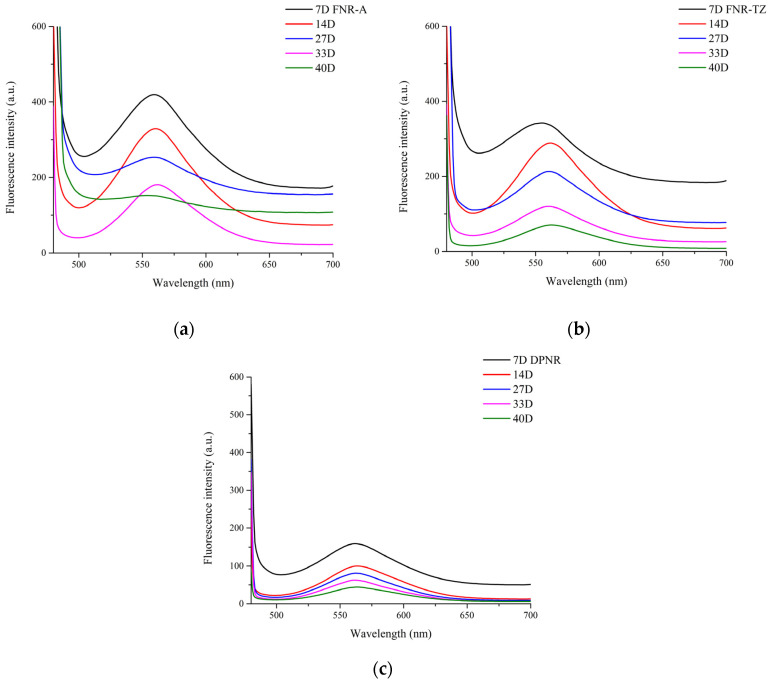
Fluorescence spectra of (**a**) FNR-A, (**b**) FNR-TZ, and (**c**) DPNR latexes at various storage times.

The fluorescence images captured by CLSM of the fluorescence dye labelled specifically for proteins are shown in Figure 1, where green represents tagged proteins. These fluorescence images reveal the higher protein content of FNR samples compared to DPNR due to the reduction in the number of proteins after deproteinization. The changes in protein contents during prolonged storage of latex were determined by fluorescence spectra recorded using a JASCO FP-6200 spectrofluorometer. Figure 2 presents the fluorescence spectra of (a) FNR-A, (b) FNR-TZ, and (c) DPNR over a storage period of up to 40 days. The spectra were obtained using Qubit protein dye (with baseline compensation) and analyzed via UV–vis spectroscopy at a wavelength (λ) range of 450–700 nm. Among these samples, FNR-A latex exhibited the highest number of dye-binding particles compared to the FNR-TZ and DPNR latexes. A notable trend observed was the decrease in detectable signal intensities with the progression of storage time. This indicates that there are continuous changes in protein composition with storage. However, an exception was observed for the FNR-A latex at 40 days, with a lower intensity than that at 33 days.

**Figure 3:**
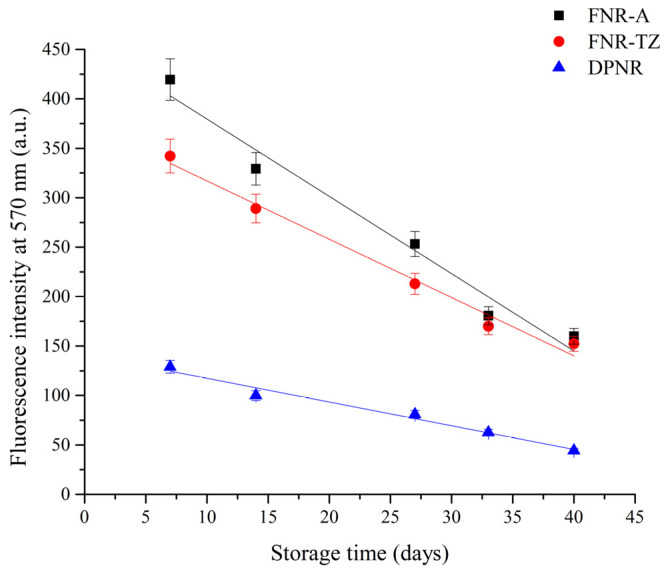
Average fluorescence emission intensity of the proteins on the rubber particles of FNR-A, FNR-TZ, and DPNR latexes at λ 570 nm, as a function of storage time.

Figure 3 illustrates the mean fluorescence emission intensity of the proteins on the rubber particles of FNR-A, FNR-TZ, and DPNR latexes at a wavelength of 570 nm, plotted against storage time. The fluorescence emission intensity of FNR-A decreased linearly with the extension of the storage time. This result suggests a more pronounced and faster reduction in the amount of rubber molecules linked to the proteins in FNR-A latex compared to FNR-TZ and DPNR latexes. This is also reflected in the lesser decreases in intensity of the latter two latexes. This observation underscores the influence of ammonia on the hydrolysis of NR molecules linked to the proteins.

### 3.2. Latex Stability

**Figure 4:**
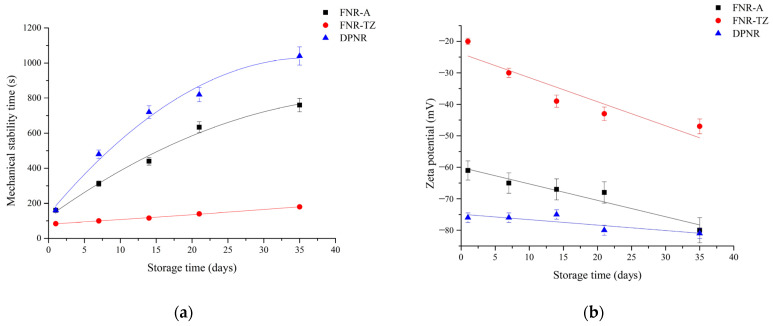
(**a**) Mechanical stability time and (**b**) zeta potential as a function of storage time for FNR-A, FNR-TZ, and DPNR latexes.

The mechanical stability results (Figure 4a) show a progressive increase in the stability of FNR-A and DPNR latexes with storage. These results, when considered in conjunction with the much higher negative zeta potential for FNR-A latex presented in Figure 4b, are consistent with the logical explanation that the stability of latex particles is controlled by their surface charge (or potential), and the higher the negative charge, the more stable the latex is going to be. This concurs with the fact that more hydrolysis of proteins linked to the rubber molecules, together with concurrent hydrolysis of phospholipids by ammonia, will generate more anionic species, and more of these will reabsorb onto the latex particles, increasing their negative zeta potential. The hydrolysis also contributes to changes in the protein composition of the latex over time. In the case of DPNR, which exhibited the highest stability and the largest negative zeta potential, this is due to the large amount of non-ionic surfactants added to the latex to maintain its stability after removing the original rubber proteins. There is no protein hydrolysis in DPNR latex. The zeta potential of the FNR-TZ latex was also found to be the least negative and the least stable of the three latexes. This reinforces the importance of protein composition from hydrolysis in the effect of ammonia on latex stability during extended storage.

Figure 4b presents the zeta potentials of FNR-A, FNR-TZ, and DPNR latexes over various storage periods. The findings from both the MST and zeta potentials indicate a consistent pattern. In the latex sample containing hydrolyzable lipids and a high ammonia content (FNR-A), the mechanical stability, hydrolyzed lipids, and surface potential all demonstrated an increase over time. The negative values observed for the zeta potential, which increased with the storage duration, underscored the anionic nature of the hydrolysis products. Conversely, when the quantity of ammonia added was minimal (FNR-TZ), the mechanical stability remained constant, and the zeta potential was found to be the least negative. The high magnitude of the zeta potentials observed in the DPNR latex could be attributed to the non-ionic surfactant employed during sample preparation. However, the consistent values observed suggest that no hydrolysis occurred in the DPNR latex.

### 3.3. Proteins Hydrolysis

**Figure 5:**
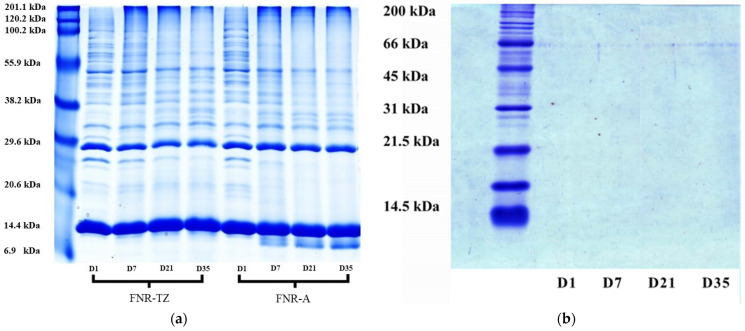
SDS–PAGE images (15% gel) of proteins extracted from (**a**) FNR-A and FNR-TZ latexes and (**b**) DPNR latex stored for 1, 7, 21, and 35 days.

Figure 5a illustrates the proteins extracted from various types of latex analyzed using the SDS-AGE technique. This method, known for its specific application in separating protein mixtures based on molecular weight differences, was used to separate extracted proteins from FNR-A and FNR-TZ latexes after storing them for 1, 7, 21, and 35 days. The results revealed the presence of protein bands with sizes ranging from 6 to over 200 kDa compared to standard proteins. These prominent protein bands were primarily derived from B- and C-sera and rubber particles [30]. The B-serum, released from the lutoids when ruptured by ammonia, was a notable component. Proteins with molecular weights above 30 kDa were predominantly from the serum fraction. In contrast, the lower-molecular-weight proteins were associated with the rubber fraction.

Certain protein bands were similar to small rubber particle protein (SRPP, 29 kDa) and rubber elongation factor (REF, 14.5 kDa), two known allergenic proteins [30,31,32,33]. In the case of FNR-TZ, protein bands within the 50 to 100 kDa and 20 to 26 kDa ranges gradually diminished in color intensity during storage, as shown in Table 2. In comparison, bands at 36 kDa and 10 kDa emerged over a storage period of up to 35 days. The color intensity of low-molecular-weight protein bands increased from 6.911 to 17.325. This result suggests that proteins with molecular weights of around 50 to 100 kDa and 20 to 26 kDa were fragmented into smaller units, even when the latex was stored in an ultralow-ammonia environment. For the FNR-A latex, protein degradation was noticeably faster, with a distinct 8 kDa protein band present compared to that of FNR-TZ. This difference demonstrates that increased ammonia concentration results in faster breakdown of high-molecular-weight NR proteins into smaller fragments or oligopeptides. Previous studies have reported that these oligopeptides, once adsorbed onto the surface of NR particles, can enhance the stability of NR latex due to their charged nature [27].

Two primary protein bands at 14 (REF, Hev b1) kDa and 25 (SRPP, Hev b3) kDa were present in FNR-A and FNR-TZ over the entire storage duration up to 35 days. Table 2 shows a slight change in the Hev b1 and Hev b3 color intensity after storing FNR-A latex for 35 days. This shows that REF and SRPP are both resistant to ammonia-induced degradation via hydrolysis [33,34]. Both Hev b1 and b3 proteins were found to adhere to the surface of NR particles through specific attachments. The Hev b1 protein was incorporated into the non-rubber layers surrounding large rubber particles.

In contrast, Hev b3 was bound to the surface of small rubber particles [35]. Both Hev b1 and Hev b3 are allergenic proteins that can withstand protein hydrolysis by ammonia. This finding corroborates that NR latex, even after being stored in high levels of ammonia for an extended period, still contains a small number of allergenic proteins. Therefore, these proteins need to be removed for product safety considerations.

Figure 5b illustrates the SDS-PAGE of extracts from DPNR latex kept for 1, 7, 21, and 35 days. Remarkably, no protein bands were found after 35 days of prolonged storage. This shows that no new proteins were generated by hydrolysis in the DPNR latex after the sample was first prepared. There were no residual proteins in this latex.

### 3.4. Effects of Ammonia on the Latex Film Morphology Arising from Storage Duration and Hydrolysis

The effects of non-rubber components on the surface of NR particles affecting NR latex film formation have been reported previously. The presence of a non-rubber mixed layer of proteins and long-chain fatty acid (LCFA) soaps surrounding the NR particles impedes particle deformation during film formation [36]. This impedes the flattening of the rubber particles and, hence, retards the rate of film formation.

**Figure 6:**
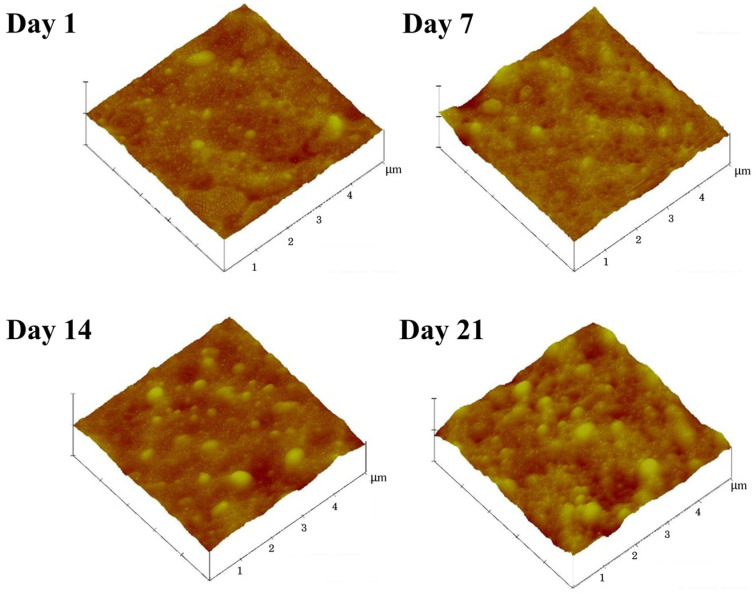
Surface morphology of latex films prepared from FNR-A latex and stored for 35 days. The films were examined 1, 7, 14, and 21 days after dipping.

**Figure 7:**
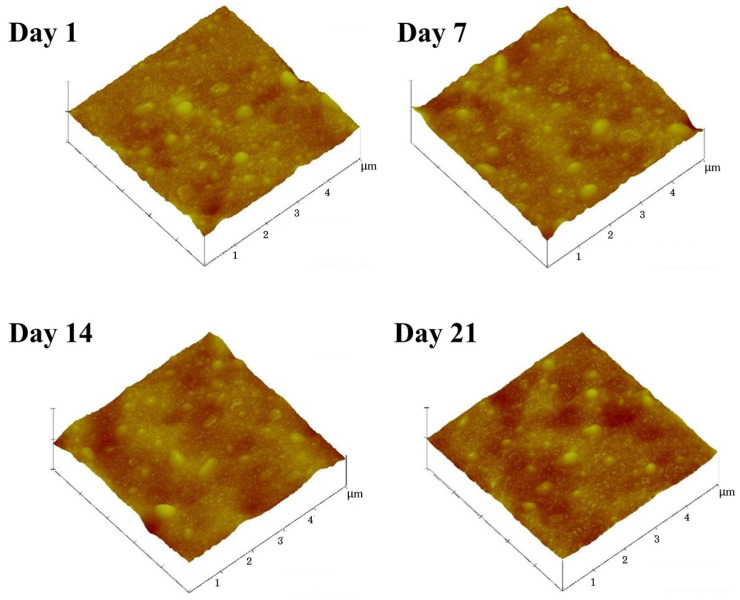
Surface morphology of latex films prepared from FNR-TZ latex stored for 35 days. After dipping, the films were examined after 1, 7, 14, and 21 days.

**Figure 8:**
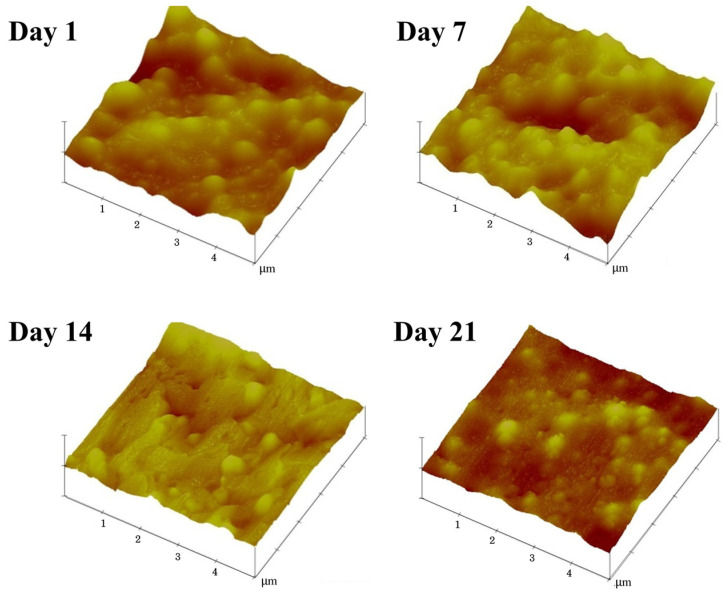
Surface morphology of latex films prepared from DPNR latex and stored for 35 days. After dipping, the films were examined after 1, 7, 14, and 21 days.

**Figure 9:**
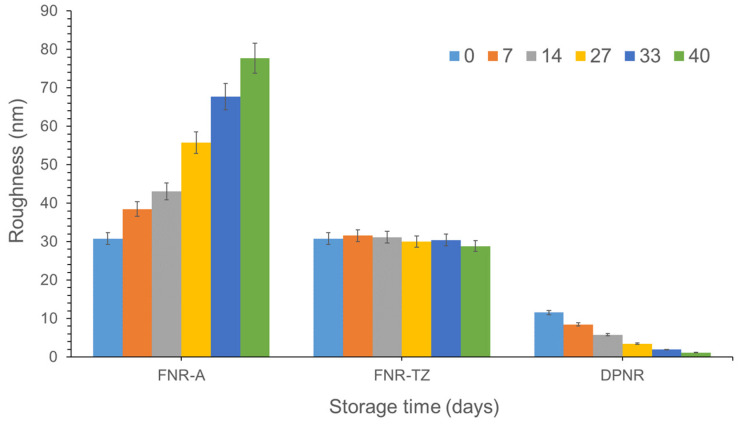
Surface roughness of the NR latex films prepared from FNR-A, FNR-TZ, and DPNR latexes stored for various durations.

In the study of the film formation and film morphology, two different sets of storage methods were used. In the first method, FNR-A, FNR-TZ, and DPNR latexes were stored in the dark for 35 days to undergo hydrolysis, then dip-coated on glass plates, and subsequently the dip-coated films were kept for 1 to 21 days prior to AFM analysis. The aim was to study the effects of hydrolyzed proteins (by ammonia for 35 days) on the aging of latex films. The results are shown in Figure 6, Figure 7 and Figure 8. In the second set of experiments, the samples were dipped in latex stored for a specific number of days (from 0 to 40 days), and then the film formation process was observed by AFM immediately from the nascent stage. The objective was to determine the effect of aging (storage) on the latex film and the accompanying evolution of the surface roughness profile of the resulting dipped films with time. This second set of experiments indirectly examined the effects of the extent of protein hydrolysis and the accompanying protein composition changes on the film formation process and kinetics (Figure 9).

**Scheme 1:**
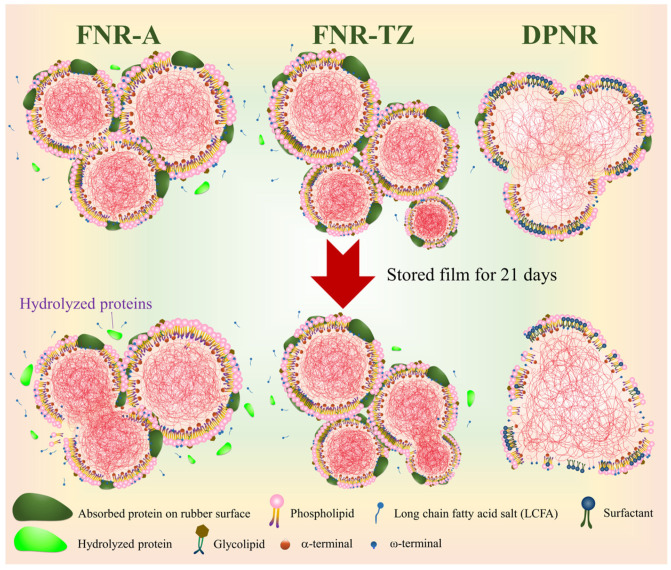
Proposed film formation mechanism of FNR-A, FNR-TZ, and DPNR during storage film for 21 days.

Figure 6 depicts the surface morphology of FNR-A latex films stored for 35 days and visualized on days 1, 7, 14, and 21 after the film formation. On days 1 and 7, the surface morphology of the FNR-A films treated with 0.6% ammonia was visually smooth; only a few individual small rubber particles were visible. Rubber particles of varying sizes began to emerge randomly from the surface of the film after 14 days of storage. By this point, the surface was no longer smooth but resembled the surface relief of mountains and valleys. Approximately 27.81% of the film surface was covered with large rubber particles (LRPs). In contrast, hardly any small rubber particles (SRPs) were visible. After that, on day 21, the number of LRPs increased, occupying 45.38% of the area. During storage at high ammonia concentrations, lipids and proteins undergo hydrolysis, breaking down into smaller molecules or fragments [18]. Some non-rubber component (NRC) layers could not form completely, as shown for FNR-TZ (see Figure 7 below). Nonetheless, this persisted, since FNR latex still retains numerous residual NRCs. A logical explanation is that after 14 days, some partial interparticle diffusion between rubber particles forms the surface relief resembling mountains and valleys. The presence of residual NRCs impeded the interparticle diffusion; hence, the FNR-A film was not smooth. The proposed mechanism is illustrated in Scheme 1.

In contrast, the surface morphology of latex films from low-ammonia FNR-TZ latex (Figure 7) was markedly distinct from that of FNR-A films. On day 21, it was seen that the surface of the FNR-TZ films was fairly smooth. Since the NRC mixed layer around the NR particles impedes the coalescence of the latex particles and the diffusion of molecular chains during film formation, very few individual large NR particles were visible on the surfaces of the FNR-TZ films, as proposed in Scheme 1.

The surface morphology of latex films prepared from DPNR latex and stored for 35 days was viewed on days 1, 7, 14, and 21 after preparation (Figure 8). The morphology of DPNR was distinct from that of the other two latexes. The surfaces were tacky, and right from the first day the films were merged without visible holes. The mixed layer of protein and LCFA soaps surrounding the latex particles in DPNR latex has been removed during deproteinization and replaced by non-ionic surfactants. Without this mixed layer surrounding the latex particles, the diffusion of rubber molecules between particles will be unimpeded, facilitating the coalescence of latex particles to form a continuous and smooth film [36].

Figure 9 shows the surface roughness of the latex films prepared from FNR-A, FNR-TZ, and DPNR latexes stored for 0, 7, 14, 27, 33, and 40 days before the latex was used for the observation of film formation behavior from the nascent stage. The roughness of the film surface of the FNR-A latex increased with the storage duration of the latex at the fastest rate, reaching a height of almost 80 nm. These findings are in agreement with those shown in Figure 6. The surface film roughness of the FNR-TZ latex was essentially constant, at about 30 nm. In contrast, the DPNR film had the lowest roughness value of about 10 nm, and its roughness decreased rapidly, with a faster flattening rate, to less than 2 nm as the storage time of the latex increased. The AFM results show that changes in the protein composition of different latex types brought about by ammonia profoundly affect the stability and film formation behavior of the latex.

## 4. Conclusions

This work has demonstrated that a combination of fluorescence protein markers, CLSM, and SDS-PAGE techniques can successfully monitor the changes in protein composition with the storage duration of different NR latexes. The results indicate extensive hydrolysis of proteins in FNR-A and FNR-TZ latexes monitored for up to 40 days of storage after preparation. The changes in protein composition are also reflected in the amounts of ionic species generated from the hydrolysis of proteins and phospholipids, as measured by the MST and zeta potentials of the latexes throughout the duration of storage. This resulted in a concurrent increase in latex stability following the increase in negative charges generated by ammoniation of the latex. There is a linear relationship between protein hydrolysis and storage duration. This shows that proteins linked to the rubber molecules are most susceptible to ammonia hydrolysis, as reflected in the rapid decrease in proteins linked to the rubber molecules during the storage of FNR-A latex compared to FNR-TZ latex. DPNR latex is the least affected by protein hydrolysis in ammonia, since most proteins have already been removed during the preparation stage. This shows that protein hydrolysis depends on both ammonia and protein concentrations, resulting in different extents of changes in the protein composition of the three latexes. High-molecular-weight proteins were broken down into smaller fragments through hydrolysis in the presence of ammonia. This is reflected in the changes in the protein composition, as confirmed by SDS-PAGE. Together, these techniques provided important information on the spatial distribution of the residual allergenic proteins. This, in turn, will facilitate the design of a better leaching protocol for the removal of residual proteins to produce low-allergenic latex-dipped products. This work shows that changes in protein composition with storage in ammonia strongly impact the morphology of thin latex films, as their morphology depends on the types and amounts of proteins present in the latex. The AFM results corroborated this, showing that the film roughness was the highest for FNR-A, unchanged for FNR-TZ, and decreased for DPNR latexes following prolonged hydrolysis.

## Figures and Tables

**Table 1:** The mean diameter, protein content, and linked fatty acid ester contents of FNR-A, FNR-TZ, and DPNR latexes.

Samples	Mean Diameter (nm)	Protein Content (wt.%)	Ester Content (mmol/kg Rubber)
FNR-A	636 ± 2	4.30 ± 0.02	28.1 ± 0.2
FNR-TZ	635 ± 1	4.63 ± 0.03	28.5 ± 0.5
DPNR	664 ± 3	0.05 ± 0.02	25.9 ± 0.7

**Table 2:** Determination of the color intensity of protein bands from the SDS-PAGE results of FNR-A and FNR-TZ.

Storage Time (Days)	Color Intensity							
	FNR-A				FNR-TZ			
	100–50 kDa	36–10 kDa	29.6 kDa (Hev b3)	14.4 kDa (Hev b1)	100–50 kDa	36–10 kDa	29.6 kDa (Hev b3)	14.4 kDa (Hev b1)
1	45.996	11.077	151.412	180.345	38.056	6.911	156.813	181.712
35	37.402	59.008	147.836	177.424	39.763	17.325	131.313	178.529

## Data Availability

Data are contained within the article.

## References

[1] Xiang Q., Xia K., Dai L., Kang G., Li Y., Nie Z., Duan C., Zeng R. (2012). Proteome analysis of the large and the small rubber particles of *Hevea brasiliensis* using 2D-DIGE. Plant Physiol. Biochem..

[2] Tuampoemsab S., Nimpaiboon A., Sakdapipanich J.T. (2015). Quantitative Analysis of Isoprene Units in Natural Rubber and Synthetic Polyisoprene using ^1^H-NMR Spectroscopy with an Internal Standard. Polym. Test..

[3] Sansatsadeekul J., Sakdapipanich J., Rojruthai P. (2011). Characterization of Associated Proteins and Phospholipids in Natural Rubber Latex. J. Biosci. Bioeng..

[4] Cornish K., Wood D.F., Windle J.J.J.P. (1999). Rubber particles from four different species, examined by transmission electron microscopy and electron-paramagnetic-resonance spin labeling, are found to consist of a homogeneous rubber core enclosed by a contiguous, monolayer biomembrane. Planta.

[5] Cornish K. (2001). Biochemistry of natural rubber, a vital raw material, emphasizing biosynthetic rate, molecular weight and compartmentalization, in evolutionarily divergent plant species. Nat. Prod. Rep..

[6] Wood D.F., Cornish K. (2000). Microstructure of Purified Rubber Particles. Int. J. Plant Sci..

[7] Tarachiwin L., Sakdapipanich J., Ute K., Kitayama T., Tanaka Y. (2005). Structural Characterization of α-Terminal Group of Natural Rubber. 2. Decomposition of Branch-Points by Phospholipase and Chemical Treatments. Biomacromolecules.

[8] Mekkriengkrai D., Sakdapipanich J.T., Tanaka Y. (2006). Structural Characterization of Terminal Groups in Natural Rubber: Origin of Nitrogenous Groups. Rubber Chem. Technol..

[9] Fu X., Huang C., Zhu Y., Huang G., Wu J. (2019). Characterizing the naturally occurring sacrificial bond within natural rubber. Polymer.

[10] Qu W., Zhu Y., Huang G., Huang C., Luo M.-C., Zheng J. (2016). Study of molecular weight and chain branching architectures of natural rubber. J. Appl. Polym. Sci..

[11] Liu J., Wu S., Tang Z., Lin T., Guo B., Huang G. (2015). New evidence disclosed for networking in natural rubber by dielectric relaxation spectroscopy. Soft Matter.

[12] Yamashita S., Yamaguchi H., Waki T., Aoki Y., Mizuno M., Yanbe F., Ishii T., Funaki A., Tozawa Y., Miyagi-Inoue Y. (2016). Identification and reconstitution of the rubber biosynthetic machinery on rubber particles from *Hevea* brasiliensis. eLife.

[13] Berthelot K., Lecomte S., Estevez Y., Zhendre V., Henry S., Thévenot J., Dufourc E.J., Alves I.D., Peruch F. (2014). Rubber particle proteins, HbREF and HbSRPP, show different interactions with model membranes. Biochim. Biophys. Acta Biomembr..

[14] Oouchi M., Ukawa J., Ishii Y., Maeda H. (2019). Structural Analysis of the Terminal Groups in Commercial *Hevea* Natural Rubber by 2D-NMR with DOSY Filters and Multiple-WET Methods Using Ultrahigh-Field NMR. Biomacromolecules.

[15] Lowe J.S. (1959). Formation of volatile fatty acids in ammonia preserved natural latex concentrate. Trans. Instn. Rubb. Ind..

[16] McGavack J. (1947). Proper Preservation and Storage of Latex. Rubber Chem. Technol..

[17] Blackley D.C. (1997). Polymer Latices.

[18] Kumarn S., Churinthorn N., Nimpaiboon A., Sriring M., Ho C.-C., Takahara A., Sakdapipanich J. (2018). Investigating the Mechanistic and Structural Role of Lipid Hydrolysis in the Stabilization of Ammonia-Preserved *Hevea* Rubber Latex. Langmuir.

[19] Chen S.-F., Ng C.-S. (1984). The Natural Higher Fatty Acid Soaps in Natural Rubber Latex and Their Effect on the Mechanical Stability of the Latex. Rubber Chem. Technol..

[20] Hasma H. (1991). Lipids associated with rubber particles and their possible role in mechanical stability of latex concentrates. J. Nat. Rubber Res..

[21] Liu X.-X., He M.-F., Luo M.-C., Wei Y.-C., Liao S. (2022). The role of natural rubber endogenous proteins in promoting the formation of vulcanization networks. e-Polymers.

[22] O’Hehir R.E., Sutherland M.F., Drew A.C., Rolland J.M. (2008). Latex Allergy. Allergy and Allergic Diseases.

[23] Sussman G.L., Beezhold D.H., Kurup V.P. (2002). Allergens and natural rubber proteins. J. Allergy Clin. Immunol..

[24] Kahn S.L., Podjasek J.O., Dimitropoulos V.A., Brown C.W. (2016). Natural rubber latex allergy. Dis. Mon..

[25] Mak T.W., Saunders M.E., Mak T.W., Saunders M.E. (2006). 28—Allergy and Hypersensitivity. The Immune Response.

[26] Yeang H.Y., Arif S.A.M., Yusof F., Sunderasan E. (2002). Allergenic proteins of natural rubber latex. Methods.

[27] Ho C.C., Kondo T., Muramatsu N., Ohshima H. (1996). Surface Structure of Natural Rubber Latex Particles from Electrophoretic Mobility Data. J. Colloid. Interface Sci..

[28] Wu J., Qu W., Huang G., Wang S., Huang C., Liu H. (2017). Super-Resolution Fluorescence Imaging of Spatial Organization of Proteins and Lipids in Natural Rubber. Biomacromolecules.

[29] Nipithakul T., Watthanachote L., Kalapat N. (2012). Preliminary study of extractable protein binding using maleic anhydride copolymer. Chin. J. Polym. Sci..

[30] Elumalai S., Hamid S., Cardosa M.J., Yeang H.Y. (1994). Allergenic Proteins of *Hevea brasiliensis* Latex Fractions. J. Rubber Res..

[31] Chen Z., Cremer R., Posch A., Raulf-Heimsoth M., Rihs H.-P., Baur X. (1997). On the allergenicity of Hev b 1 among health care workers and patients with spina bifida allergic to natural rubber latex. J. Allergy Clin. Immunol..

[32] Lu L.J., Kurup V.P., Hoffman D.R., Kelly K.J., Murali P.S., Fink J.N. (1995). Characterization of a major latex allergen associated with hypersensitivity in spina bifida patients. J. Immunol..

[33] Yeang H.Y., Cheong K.F., Sunderasan E., Hamzah S., Chew N.P., Hamid S., Hamilton R.G., Cardosa M.J. (1996). The 14.6 kd rubber elongation factor (Hev b 1) and 24 kd (Hev b 3) rubber particle proteins are recognized by IgE from patients with spina bifida and latex allergy. J. Allergy Clin. Immunol..

[34] Dennis M.S., Light D.R. (1989). Rubber elongation factor from *Hevea brasiliensis*: Identification, characterization, and role in rubber biosynthesis. J. Biol. Chem..

[35] Berthelot K., Lecomte S., Estevez Y., Coulary-Salin B., Bentaleb A., Cullin C., Deffieux A., Peruch F. (2012). Rubber elongation factor (REF), a major allergen component in *Hevea brasiliensis* latex has amyloid properties. PLoS ONE.

[36] Wei Y.-C., Xia J.-H., Zhang L., Zheng T.-T., Liao S. (2020). Influence of non-rubber components on film formation behavior of natural rubber latex. Colloid. Polym. Sci..

